# Minimal invasive posterior correction of Lenke 5C idiopathic scoliosis: comparative analysis of minimal invasive vs. open surgery

**DOI:** 10.1007/s00402-019-03166-y

**Published:** 2019-03-14

**Authors:** Wiktor Urbanski, Rafał Zaluski, Anis Kokaveshi, Silvester Aldobasic, Grzegorz Miekisiak, Piotr Morasiewicz

**Affiliations:** 1 Department of Orthopedics and Traumatology, Wrocław Medical University, ul. Borowska 213, 50-556 Wrocław, Poland; 2 Department of Neurosurgery, Wrocław Medical University, Wrocław, Poland; 3 Institute of Medicine, University of Opole, Opole, Poland

**Keywords:** Idiopathic scoliosis, Minimally invasive surgery, Posterior spinal fusion, Navigation

## Abstract

**Introduction:**

Surgical management of adolescent idiopathic scoliosis in spite of usually favourable outcomes is still a major operation. Therefore, efforts are being undertaken to minimalize the procedure, reduce the surgical trauma and postoperative convalescence. The study was designed to compare posterior minimal invasive surgery using navigation based on intraoperative 3D imaging and standard open instrumented fusion in Lenke 5C idiopathic scoliosis treatment.

**Materials and methods:**

From eight patients with Lenke 5C curves planned for posterior correction and instrumented fusion, four were treated with minimally invasive and four had open procedure. Operation length, estimated blood loss, number of fusion levels, days of opioid intake, length of hospital stay and radiation doses required were noted. Radiographic assessment of spinal curvatures was performed (magnitude, flexibility, sagittal alignment). The comparison of the data was done between open and minimally invasive treated patients.

**Results:**

In minimally invasive surgery group, the operations were longer on average 285 min ± 47.5 than in the open surgery group, 242.5 min ± 44.5 (*p* = 0.371) and resulted in slightly inferior coronal curve correction by 68.25% ± 6.2 vs. 78.25% ± 8.8, respectively (*p* = 0.072). We observed a clear reduction of intraoperative blood loss in minimally invasive patients (mean 138.75 ± 50 vs. 450 ± 106 ml, *p* = 0.016), shorter hospital stay, average 3.75 vs. 7 days (*p* = 0.043) and lower opioid requirements postoperatively − 2 vs. 3.25 days (*p* = 0.015).

**Conclusions:**

The minimally invasive approach to idiopathic scoliosis treatment is a very promising technique to limit the extent of surgery maintaining the same goals as in the open method. It allows for lower blood loss, less requirement for opioids and a shorter hospital stay.

## Introduction

Open instrumented posterior spinal fusion of progressive adolescent idiopathic scoliosis may be considered as the standard management [1, 2]. The results of the procedure are usually satisfactory from the patient’s but also from parent or surgeon’s point of view. However, this is a still major surgical procedure with a significant rate of blood transfusions, surgical site infection and lengthy hospital stay [2]. Additionally, there are concerns regarding the condition of paraspinal muscles after dissection especially in long-term perspective—scarring, muscle morbidity from denervation with subsequent atrophy and reduced extension of the trunk [3–5]. Therefore, efforts to limit the extent of surgical trauma have been undertaken. Since 2011, reports have been emerging regarding posterior minimally invasive procedures in idiopathic scoliosis, however, follow ups are short and data obtained so far have been rather scanty [6–9]. There is an uncertainty whether benefits outweigh the efforts, risk and costs of the procedure and how advantageous the minimally invasive procedure is comparing to open techniques in idiopathic scoliosis. At present, the questions regarding the effectiveness of deformity correction, the risk of the procedure, learning curve and how it affects perioperative care, like length of hospital, need for transfusions, opioids intake remain unanswered.

The objectives of the study were to assess benefits rising from the limited surgical approach using navigation based on 3D image obtain intraoperatively in comparison to open posterior instrumented fusion in Lenke 5C idiopathic scoliosis treatment.

## Materials and methods

This is a retrospectively evaluated, prospectively collected data of patients with Lenke 5C adolescent idiopathic scoliosis, treated surgically by a single surgeon (WU) in Department of Orthopedics and Traumatology, Wrocław Medical University. Four consecutive patients treated operatively with posterior minimally invasive procedure in 2018 were compared to four consecutive patients treated with open posterior spinal fusion between 2016 and 2017.

The authors reported the operation length, estimated blood loss (EBL), extent of fusion, number of fusion levels, and details of postoperative care such as days of opioids intake, as well as the length of hospital stay. The spinal curves were assessed, along with their flexibility, magnitude, but also the spinal sagittal profile prior and after the operation. Radiation doses required for open and minimally invasive procedures were noted. The comparison of the data was done between open and minimally invasively treated patients.

**Fig. 1:**
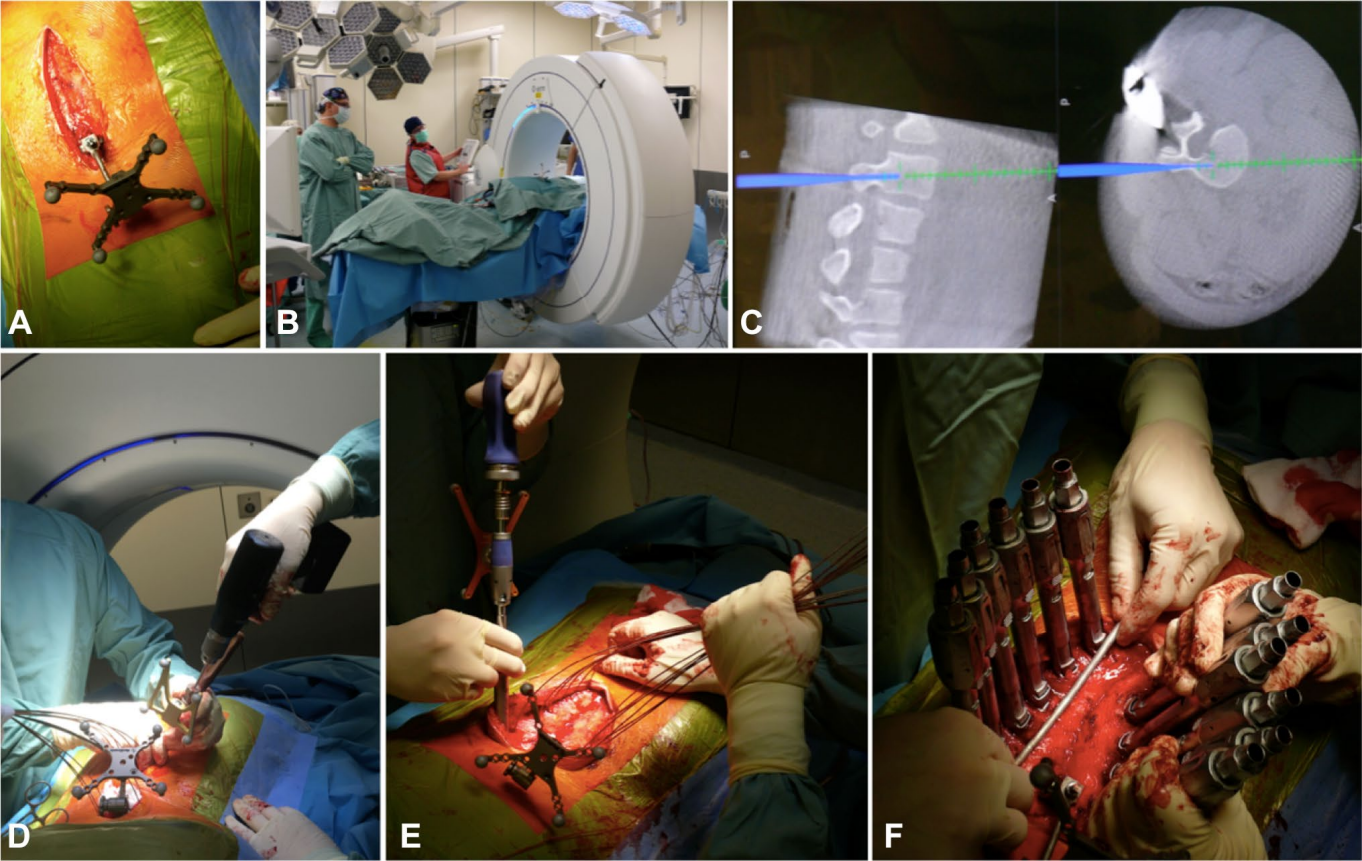
Description of minimally invasive surgery. **a** Midline skin incision with fascia exposition, mounting of reference frame on spinous process, **b** 3D scan with O-arm, **c** image from S7 navigation system, **d** stab incision in fascia and cannulising the pedicles with navigated power drill and subsequent guide wires’ insertion. **e** Screws’ insertion using guide wires and navigation, **f** screws with extenders inserted trans-fascia

Open surgery was done through standard midline posterior approach. All patients in the group had pedicle screws inserted freehanded followed by O-arm scans to assess screw position. Lower facets were removed, but otherwise no spinal release was performed. In minimally invasive surgery group, skin was incised in midline, fascia exposed and screws were inserted through stab incisions in fascia. To cannulate pedicles, the authors navigated a 3.5 mm power drill and after the guide wire insertion, lower facets were removed with a high-speed burr. The procedure was done under the guidance of navigation (Stealth Station S8 Surgical Navigation System, Medtronic) based on intraoperatively obtained 3D image with O-Arm (Medtronic) (Fig. 1).

In both groups, similar screw constructs were used with a high screw density, on the convex side CoCr 5.5 rods were used and undercontoured titanium on concavity. 3D scan with o-arm to check the screw position was performed in both groups. All operations were done with motor-evoked potential neuromonitoring (NIM Eclipse, Medtronic).

Data were presented as the mean ± standard deviation. A two-tailed paired *t* test was performed to assess variables between minimally invasive and open groups at two time points (preoperatively, postoperatively), with *p* < 0.05 considered statistically significant.

## Results

Demographic data and preoperative curve characteristics showed, that patients from open surgery group were older on average by 6 years, they had smaller (by 10^0^), but stiffer (on average by nearly 18%) curves compared to the minimally invasive group. There were differences noted in the sagittal alignment between groups. The open surgery patients had a smaller lordosis by average 10^0^, greater kyphosis by 13^0^ with SVA greater on average by 31 mm. However, none of the above data was confirmed statistically significant (Table 1).

**Table 1:** Demography and preoperative curve characteristics

	MIS	Open	*p* value
Age	15.5 ± 2.06	21.25 ± 9.98	0.438
Female/male	0/4	1/3	0.391
Coronal curve magnitude	57.25^0^ ± 10.64^0^	47^0^ ± 7.78	0.209
Coronal curve flexibility	56% ± 6.4	38.25% ± 9.01	0.051
Thoracic kyphosis	23.6 ± 7.61	37 ± 16.06	0.072
Lumbar lordosis	57.975 ± 9.31	47.85 ± 23.31	0.356
SVA [mm]	22.1 ± 9.94	53.5 ± 39.78	0.179

*MIS* minimal invasive surgery

In both groups, a similar number of levels have been fused. Open surgery provided slightly superior (by 10%) coronal curve correction than minimally invasive surgery, and a shorter surgical time (by average 43 min), but with markedly increased intraoperative blood loss (by 312 ml on average). In minimally invasive group, the implant introduction was done with navigation based on 3D scan, therefore, intraoperative radiation was higher in the group.

Minimally invasive group patients spent noticeably less time in hospital postoperatively, being discharged on average before day 4 and we observed opioid intake reduction to 2 days postoperatively, comparing to 7 day stay and 3 days of opioids in the open group.

The sagittal parameters analysed has not revealed any significant differences between the groups except for SVA change. In open surgery group, SVA was primarily bigger (on average by 7.3 mm) and reduced from 53 mm preoperatively to 30 mm postoperatively (Table 2).

**Table 2:** Comparison of surgical information and postoperative radiographic parameters

	MIS	Open	*p* value
Operation length [min]	285 ± 47.56	242.5 ± 44.51	0.371
EBL [ml]	138.75 ± 50.04	450 ± 106.06	0.016
Radiation mGy/mGy cm^2^	8.9 ± 3.9/581.8 ± 158.3	18.3 ± 13.4/411.7 ± 195.2	0.285
Number of levels fused	6.5 ± 0.86	5.75 ± 0.43	0.215
Opioid intake (days)	2 ± 0.7	3.25 ± 0.43	0.015
Length of hospital stay [days]	3.75 ± 0.433	7 ± 3	0.043
Coronal curve correction (%)	68.25 ± 6.18	78.25 ± 8.84	0.072
Thoracic kyphosis	26.075 ± 8.53	32.4 ± 12.51	0.14
Lumbar lordosis	55.625 ± 5.54	48.45 ± 9.16	0.171
SVA [mm]	22.925 ± 18.8	30.22 ± 14.47	0.641

*MIS* minimal invasive surgery, *EBL* estimated blood loos

## Discussion

**Fig. 2:**
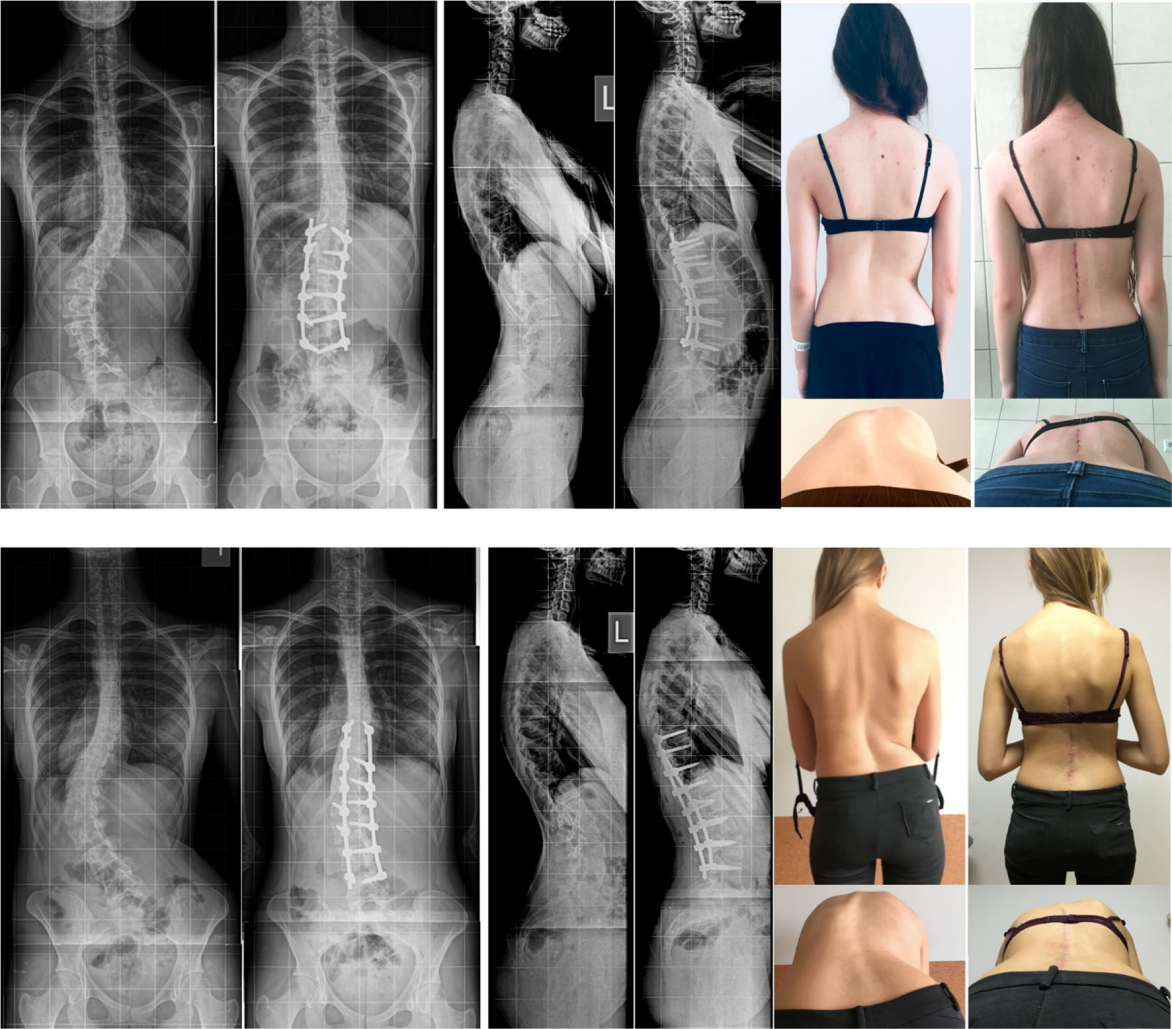
Clinical examples of minimally invasively treated idiopathic scoliosis

This is an early report describing posterior minimally invasive approach for surgical treatment of idiopathic scoliosis. We have presented the perioperative characteristics of Lenke 5 curves treated with either standard open or minimally invasive technique and we matched early results in both methods. The comparison showed significant reduction of intraoperative blood loss, opioid requirements but at the cost of lesser coronal curve correction in the minimally invasive technique. Unfortunately, this is a small series of patients, too small to formulate any strong recommendations. There were some discrepancies in the characteristics of analysed groups—in open surgery group patients were slightly older and had stiffer curves. The results presented, contain only early postoperative observations, which further contribute to the paper limitations. However, we have presented a successfully implemented technique of significant reduction of the extent of surgical intervention (Fig. 2), which was confirmed with the numbers presented. The additional advantage of the paper is an inclusion of patients with the same curve pattern—Lenke 5C.

The general benefits and disadvantages of minimally invasive spinal surgery have been well described in the literature of the 2 last decades. Numerous authors concluded advantages driven by smaller trauma in MIS procedures, bringing up shorter convalescence, less opioid intake, shorter hospital stay, comparing to open procedures [10–14]. These papers concern degenerative or trauma cases where the benefits from MIS are clear with abundant supportive literature [11–13], but the benefits of MIS in adolescent idiopathic scoliosis are not so obvious, with a few reports focusing on the topic [6–9, 15–17]. Most of the reports consistently demonstrated similarly to our results, reduction of intraoperative blood loss [9, 16, 17], need for pain medications [6, 9] and shorter hospital stay [6, 7, 17] but on the other hand inferior coronal curve correction [17] and longer operation length [9, 15–17]. We believe that longer operation time is partially related to steep learning curve, however, even in a proficient surgeon’s hand, the procedure might be slightly longer than the standard approach. Radiographic assessment showed lower coronal correction in the minimally invasive group, but the differences were minor, and according to Miyanji et al. it did not shown clinical relevance [17].

Sarwahi et al. in their experience did not observe any advantages in perioperative care between MIS and open surgery patients [15]. They reported longer surgical time in MIS group, similar intraoperative blood loss, and similar hospital stay in both methods. However, the described technique is more “mini open”, requiring muscles dissection using the Wiltse approach to the facets. Whilst we presented a truly minimally invasive technique, similar to percutaneous fixation seen in trauma with only extension to ostetomize facet joints and graft insertion to obtain fusion and one midline skin incision for cosmetic reasons. We believe that we presented a truly minimally invasive approach, which allowed for significant surgical trauma reduction with all its consequences.

Radiation necessary for the presented procedure might raise concerns, as it was previously observed that navigation required more radiation than other methods [18]. Although in this series we have noticed increased radiation and the difference has not been significant, possibly due to the fact that patients having a open procedure had a O-arm scan to assess pedicle screws position and minimally invasive patients had only scans necessary for navigation purposes.

We included into the study patients with Lenke 5C who required fusion only in lower thoracic and lumbar spine. The pedicles at these levels are fairly wide and rather easier for percutaneous procedures, unlike to thoracic spine, especially at the concave side where pedicles are often narrow and sclerotic. So far in our institution it is considered as a method limitation—to implement the method we need flexible curve and wide pedicles, however, we will soon extend indications to other curve types, switching if necessary to constructs with high screw density on convexity if concave pedicle would not allow for safe screw introduction.

The controversy of the idea of posterior minimally invasive procedures in adolescent idiopathic scoliosis is due to the fact that open procedures are mostly very successful with low morbidity and good outcome [1, 2]. The question may be asked why to change the current practice, what is the point to change what works good. Is “better the enemy of good” in this particular case? We believe that the saying does not apply here. We believe that any reduction of surgical trauma should be taken into consideration, moreover our early results suggest that the approach limits trauma concomitantly allowing for the good outcome.

Summarising available literature and our own experience, there is still insufficient evidence regarding benefits of minimally invasive operation in idiopathic scoliosis in comparison to standard open management. Little is known about the indications—what curve type, magnitude, flexibility or patients’ age would be the best indication for the minimally invasive approach. Another question concerns technical difficulties that arise while trying to perform properly all necessary elements of the procedure—screw insertions, facetectomies and also steep learning curve to master the technique. Further research is required to answer the problems mentioned above, especially since the idea of minimally invasive management of scoliosis seems to be very promising.
